# Nonvolatile modulation of electronic structure and correlative magnetism of L1_0_-FePt films using significant strain induced by shape memory substrates

**DOI:** 10.1038/srep20199

**Published:** 2016-02-01

**Authors:** Chun Feng, Jiancheng Zhao, Feng Yang, Kui Gong, Shijie Hao, Yi Cao, Chen Hu, Jingyan Zhang, Zhongqiang Wang, Lei Chen, Sirui Li, Li Sun, Lishan Cui, Guanghua Yu

**Affiliations:** 1 Department of Materials Physics and Chemistry, University of Science and Technology Beijing, Beijing 100083, People’s Republic of China; 2 State Key Laboratory of Heavy Oil Processing, China University of Petroleum-Beijing, Beijing 102249, China; 3 Centre for the Physics of Materials and Department of Physics, McGill University, Montreal, Quebec H3A2T8, Canada; 4 Department of Mechanical Engineering and Texas Center for Superconductivity (TcSUH), University of Houston, Houston, Texas 77204, USA

## Abstract

Tuning the lattice strain (*ε*_L_) is a novel approach to manipulate the magnetic, electronic, and transport properties of spintronic materials. Achievable *ε*_L_ in thin film samples induced by traditional ferroelectric or flexible substrates is usually volatile and well below 1%. Such limits in the tuning capability cannot meet the requirements for nonvolatile applications of spintronic materials. This study answers to the challenge of introducing significant amount of elastic strain in deposited thin films so that noticeable tuning of the spintronic characteristics can be realized. Based on subtle elastic strain engineering of depositing L1_0_-FePt films on pre-stretched NiTi(Nb) shape memory alloy substrates, steerable and nonvolatile lattice strain up to 2.18% has been achieved in the L1_0_-FePt films by thermally controlling the shape memory effect of the substrates. Introduced strains at this level significantly modify the electronic density of state, orbital overlap, and spin-orbit coupling (SOC) strength in the FePt film, leading to nonvolatile modulation of magnetic anisotropy and magnetization reversal characteristics. This finding not only opens an efficient avenue for the nonvolatile tuning of SOC based magnetism and spintronic effects, but also helps to clarify the physical nature of pure strain effect.

Magnetic^1^,^2^,^3^,^4^, electronic^5^,^6^,^7^, and coupled magneto-transportation^8^,^9^,^10^,^11^,^12^,^13^,^14^,^15^,^16^,^17^,^18^,^19^ properties of spintronic materials and heterostructures are sensitive to their intrinsic and/or interfacial electronic structures. Studies have shown that spin distribution, charge density, orbital occupancy, and spin-orbit coupling (SOC) strength of these materials can be modified through a local electrical field or a spin polarized current, resulting in tunibility in magnetic responses^1^,^2^,^3^,^4^, magnetization switching^8^,^9^,^10^,^11^, and spin dependent transport^6^,^12^,^13^,^14^,^18^ properties. However, most of the electrical modulation methods are volatile thus not suitable for nonvolatile applications. And it is still challenging to realize effective and nonvolatile modulation of electronic and related characteristics in spintronic materials.

Lattice strain (*ε*_L_) is known to be closely related to the physical or chemical properties of a material. It has been shown by *ab initio* calculation that when the lattice constant of a semiconductor changes by 1%, there can be a change in bandgapas as much as 100 meV^20^. Thus, there is increasing interest in using lattice strain to manipulate spintronic materials properties^15^,^19^,^21^,^22^,^23^,^24^,^25^,^26^,^27^,^28^,^29^,^30^,^31^,^32^,^33^. Recent studies show that by incorporating piezo/ferroelectric materials as substrates, it is possible to use external electric field to influence the magnetic properties of deposited magnetic films through the inverse magnetoelectric coupling effect^21^,^22^,^23^,^24^,^25^,^26^. However, such strain introduction is volatile unless the magnetic domain structures can be altered^25^,^26^. Furthermore, the transferrable lattice strain in such heterostructures is normally limited to be less than 0.5%^21^,^22^,^23^,^24^, which restricts the tuning effectiveness in magnetic properties. Further studies also show that when appropriate buffer layers are used, it is possible to obtain a lattice strain as large as 3% in a deposited magnetic film, but there is little tunability in film electronic structure and magnetic properties once the strain is set^27^,^28^,^29^. More recently, the use of polymer substrates with high ductility has been explored to modulate the magnetic properties of grown films. Although polymer strain can exceed 30%^30^,^31^, mismatch in Young’s modulus between magnetic film and polymer substrate limits the possible film strain to be below 1%^32^,^33^. Thus, it is of great importance to able to generate and control a significant amount of elastic strain in spintronic films so that electronic structure and related magnetic/transport properties can be tuned.

In previous work, we demonstrate the achievement of a revertible strain as large as 6.5% in a NiTi(Nb) shape memory alloy (SMA) by coupling the pseudoelasticity of NiTi matrix with the elasticity of Nb nanowires^34^. Such a large strain output has the potential to effectively modulate the lattice strain in magnetic thin films deposited on top of it. In such a system, simple thermal treatment can introduce significant shape recovery in the SMA substrate through the inverse martensitic phase transformation, and because the elastic modulus of the SMA is on the same order as many metallic films, significantly large lattice strains can be introduced in the magnetic films (as shown in the Fig. 1). This approach allows easy strain modulation in the thin film by controlling the pre-deformation and heat treatment of the SMA substrate. Moreover, the elastic lattice strain in the film is nonvolatile because the elastic deformation of substrate can be maintained in a broad temperature range (−20 °C ∼ 100 °C). Thus, this subtle heterostructure design can help to realize large, steerable, and nonvolatile elastic strains which are desirable for effectively modifying the electronic structure and magnetic property of magnetic films. To verify this hypothesis, we deposited L1_0_-FePt films on pre-stretched NiTi(Nb) SMA substrates. L1_0_-FePt alloy is a spintronic material with high magnetocrystalline anisotropy and strong SOC strength. But effective magnetism tunability in the L1_0_-FePt films via elastic strain engineering was scarcely reported due to the difficulty of introducing significant elastic strain in such large magnetocrystalline anisotropy materials. In the current study, an in-plane compressive lattice strain as high as 2.18% was obtained in the L1_0_-FePt film, leading to prominent changes in density of state (DOS) and SOC strength, and consequently resulted in noticeable and nonvolatile change in magnetic anisotropy (up to 75%).

## Results

In this work, the revertible macro-strain (*ε*_M_) of the NiTi(Nb) substrates were tuned between 0% to 5.5% through the control of the pre-deformation amount (seen in Figure S1). The samples were first studied by X-ray diffractometer (XRD) and high-resolution electron microscopy (HRTEM). The TEM local images (Fig. 2c–f) and elemental mapping pictures (Fig. 2g–k) indicate strong adhesion between the film and the substrate. Sharp interface with no apparent atomic diffusion can be observed. Such favorable interfacial condition ensures the strain transfer from the substrate to the film. Lattice strain in the FePt film is quantified using XRD spectra (Fig. 2a). All FePt samples exhibit (111) texture, implying that the (111) plane lies parallel to the film plane. When *ε*_M_ in the NiTi(Nb) substrate increases from 0% to 5.5%, the attached FePt(111) diffraction peak (2θ) gradually shifts from 41.25° to 40.95°, indicating an increasing in the (111) plane-spacing. The (111) *d*-spacing variation ratio was used to evaluate the out-of-plane tensile strain in the film, as shown by the blue line in Fig. 2b. Assuming a FePt Poisson’s ratio υ of 0.33^35^, the in-plane compressive lattice strain (*ε*_L_) in the film can be calculated, as shown by the yellow line in Fig. 2b. A maximum *ε*_L_ value of 2.18% has been achieved, which is at least 4–5 times larger than the lattice strain that can be achieved by ferroelectric substrates (<0.5%)^21^,^22^,^23^,^24^,^25^,^26^. It should be noticed that the lattice strain in the film is not uniform across the film thickness and the calculated *ε*_L_ is an average value.

Under such large elastic strain, how do magnetic properties of the film change? From the in-plane (along the strain) and out-of-plane hysteresis loop (Figures S2–S4) measurements, the dependence of the FePt effective magnetic anisotropy (*K*_eff_) on *ε*_L_ is determined and shown in Fig. 3a. Here, *K*_eff_, the energy difference when the FePt film is magnetized along the in-plane and out-of-plane directions, is used to describe magnetic anisotropy of a film. When *K*_eff_ is positive, a sample possesses the so-called perpendicular anisotropy (PMA). In this work, all of the FePt films on NiTi(Nb) substrates possess in-plane anisotropy (as shown in the Figs S2–S4,) because of the (111) texture. Figure 3a clearly shows that the magnetic anisotropy of the FePt film grown on NiTi(Nb) is very sensitive to both the lattice strain *ε*_L_ and the film thickness (*t*). Small variation in *ε*_L_ can lead to noticeable change in the *K*_*eff*_, suggesting that the modification of lattice strain is indeed an effective method to manipulate the magnetic properties of thin films. The thinner the film, the larger the tunability in FePt magnetic responses. For a 10 nm FePt thin film, *K*_*eff*_ increases from −0.8 × 10^6^ J/m^3^ at zero strain to −0.2 × 10^6^ J/m^3^ when *ε*_L_ = −2.18%. Such significant change in the magnetic anisotropy (up to 75%) was rarely reported in hard magnetic materials used for logic storage devices, which is very important for reducing device power consumption^36^. When *t* increases from 10 nm to 15 nm or 20 nm, the tunability of magnetic property decreases. Thus, the strain effect can be considered as interfacial effect which exhibits stronger effects in smaller film thickness. For a magnetic film, the total anisotropy energy includes bulk anisotropy (*K*_v_), shape anisotropy (*K*_s_ = −2πMs^2^), and interfacial anisotropy (*K*_i_), as shown in formula 1^37^:


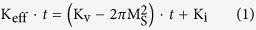


Where M_S_ is the saturation magnetization of FePt. By plotting the variation of *K*_eff_ ·*t* with *t* (Fig. 3b), we can tell from the relative contributions of *K*_v_ (the slope of the curves) and *K*_i_ (the y axis interception) to *K*_eff_. As shown in Fig. 3c, with increasing *ε*_L_, *K*_v_ only changes slightly, but *K*_i_ rises at a much higher rate. For ordered L1_0_-FePt, *K*_v_ (i.e. intrinsic magnetocystalline anisotropy) is dominated by the ordering degree of the structure^38^. Thus, it is possible that the strain has not significant influence on the ordering of the FePt atomic structure and ordering degree effect on magnetism modification is not dominant. On the other hand, the drastic increase of *K*_i_ in Fig. 3d suggests that the in-plane compressive strain in the FePt film introduces additional magnetic anisotropy with the easy magnetization direction perpendicular to the film plane, resulting in the tilting of magnetization easy axis away from in-plane direction and consequent increase of *K*_eff_. These results indicate that the *K*_eff_ manipulation observed here are mainly resulted from the interfacial anisotropy induced by the large elastic strain.

## Discussion

The interfacial strain modulation effect is hypothesized to directly link to the evolution of interfacial electronic structure of the spintronic materials. To reveal the electronic structure changes, X-ray photoelectron spectroscopy (XPS) analyses were conducted on NiTi(Nb)-SMA/L1_0_-FePt(3 nm)/Ta(5 nm) films with different amount of lattice strain. The binding energy evolution of the characteristic Fe2p and Pt4f electrons at the NiTi(Nb)/FePt interfaces, which reflects the effect of elastic strain on the interfacial electronic structure, is shown in Fig. 4. All of the Fe and Pt atoms at the interface remain in metallic states (the standard binding energy for Fe 2p_3/2_ and Pt 4f_7/2_ electrons are 706.75 eV and 70.9 eV, respectively). However, as the compressive strain increases, Fe 2p_3/2_ and Pt 4f_7/2_ peaks shift by 0.3 ∼ 0.4 eV towards the low binding energy. Actually, increasing compressive strains in the film enhances the overlap of outer electron orbits in the Fe and Pt atoms and strengthens the shielding effect to the core electrons. This is equivalent to the reduction of nuclear charge, and will consequently lead to the decline of binding energy of core electrons, and thus shifts the XPS peaks. The noticeable peak shift provides direct proof of substantial modification of the electronic structure due to elastic strain.

To theoretically reveal the elastic strain effect on electronic structures of FePt, we conducted first-principle calculations based on the density functional theory (DFT). Since we focus on the role of externally induced elastic stresses on FePt in the calculation, ideal bulk L1_0_-FePt is assumed, as shown in Fig. 5a. Considering the FePt films deposited on the NiTi(Nb) substrate are (111) oriented (seen in Fig. 2a), the elastic stress induced by NiTi(Nb) substrates is assumed to act on FePt (111) uniformly. On the other words, the [100], [010] and [001] axis of FePt crystal have the same compression ratio, seen in Fig. 5a. To show the strain effect, 4 different compressive strains, *ε*_L_ = 0%, −1%, −2%, −3%, along the three axis, were calculated.

As shown in the calculated total density of state (DOS) in Fig. 5b, the spin distribution of spin-up electrons shifts toward fermi level with respect to increasing compressive lattice strains. Typically, when *ε*_L_ = −3%, the spin distribution shift by 0.3eV. On the contrary, the spin distribution of spin-down electrons shifts away from Fermi level when we increase the straining. As a consequence, the magnetic moment of L1_0_-FePt decreases from 6.60 μ_B_ for *ε*_L_ = 0% to 6.27 μ_B_ for *ε*_L_ = −3% (seen in Figure S5b), which is consistent with our experimental observations (shown in Figure S6). Meanwhile, we also calculated the partial density of states (PDOS) of L1_0_-FePt, shown in Fig. 5c for *ε*_L_ = 0% and Fig. 5d for *ε*_L_ = −3%, respectively. For simplification, we only plot the PDOS of d orbital because it dominates near the Fermi level for both Fe and Pt atoms. As shown in Fig. 5a, the closest atoms in x-y plane is along the xy direction, so d_xy_ orbital has the largest DOS near the Fermi level (indicated by the blue line in Fig. 5c,d). Therefore, when we compress the FePt crystal along x, y and z axis, the d_xy_ orbitals have the most overlap in Fe or Pt atoms, meanwhile, the d_yz_ and d_xz_ orbitals have the most overlap between Fe and Pt atoms. Comparing with Fig. 5c, the peaks of d_xy_ and d_yz_ orbitals in Fig. 5d have the biggest drop, resulting in a largest bandwidth for d_xy_ and d_yz_ orbitals.

With increasing the bandwidth, what happens to the SOC of L1_0_-FePt systems? Here the well-known Thomas SOC formula has been used for analyses:


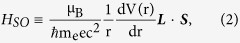


Where m_e_ is the electron mass, c is the speed of light, V(r) is the electrostatic potential at r, ***L*** and ***S*** are the orbital moment and spin moment, respectively. According to the spin-orbital matrix element analysis^39^, we project the SOC on each of the p and d orbital, plotted in Fig. 5e. Since the Pt atom is much heavier than the Fe atom, the SOC of Pt atoms dominates the total FePt SOC, and only the SOC strength (ξ) of Pt atoms is shown in Fig. 5e. As previously mentioned, with increasing compressive strain in the FePt (111) plane, the overlap of d orbitals between nearest atoms becomes larger, which reduces the localization of d orbital^40^ and results in a decrease in angular momentum *L*_*z*_ of the d orbital. From equation (2), we can see that the ξ of d orbitals will decrease with *L*_*z*_, especially for the d_xy_ orbital which have the largest overlap, as shown in Fig. 5e. On the other hand, the nearest 5p orbital of Pt is localized and does not have any overlaps, even during compression. So opposite to the d orbital, the *L*_*z*_ of 5p orbital only changes slightly during straining, leading to an increase of ξ for 5p orbitals. More importantly, the lattice compression makes the outer orbital charge density to have a quick increase. Therefore, the gradient of the electrostatic potential (dV(r)/dr) and ξ of 5p orbitals will increase considerably based on equation (2). Consequently, the ξ of 5p orbitals increases at a much faster rate than the decrease of ξ for the d orbital, which results in the total SOC strength (ξ_total_) increase with the induced in-plane strain. As seen in Fig. 5f, ξ_total_ goes up from 746 meV for *ε*_L_ = 0% to 761 meV for *ε*_L_ = −3%. In-depth study of Fig. 5e shows that p_x_ and p_y_ orbitals have much larger contributions to the increase of ξ_total_ than the other orbitals. Since p_x_ and p_y_ are in the (001) plane, and the angular moments ***L*** are perpendicular to (001) plane, i.e. along the [001] direction. Equation (2) shows that the spin moment ***S*** in the [001] orientation will have the largest SOC, leading to the largest energy level splitting along the [001] direction for FePt. As a result, when external magnetic field is applied along the [001] axis, FePt will be stabilized with the lowest total energy. On the other words, inducing the compressive strain in FePt(111) plane makes the magnetization easy axis orientated along the [001] direction and increases the magnetic anisotropic energy (MAE) of L1_0_-FePt. In Fig. 5f, when the compressive straining *ε*_L_ increases from 0% to *−*3%, the MAE of FePt system goes up from 1.68 to 
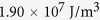
. This tendency is consistent with our experimental data of *K*_eff_ as shown in Fig. 3a.

These results indicate large and nonvolatile lattice strain (maximum of 2.18% in this paper) can be achieved in FePt films through the nonvolatile shape memory effect of the SMA substrate, which can lead to effective tuning of the interfacial electronic structures such as electronic density of state and SOC strength, and results in noticeable and nonvolatile modulations of magnetic properties. This property modulation mechanism should also be applicable to other spintronic materials because no lattice match between film and SMA substrate is required. Since many spintronic effects are closely related to the SOC strength, including the anomalous hall effect^12^,^13^,^18^, spin hall effect^8^,^14^,^15^, spin transfer torque effect^9^,^10^,^11^, magnetic proximity effect^16^,^17^, and magnetic damping effect^41^, the importance of this work is not only to open an efficient avenue for magnetism tunability of spintronic materials, but also to provide a possible opportunity for the manipulation of SOC based spintronic effects by the elastic strain engineering. It should be mentioned that much larger recoverable strain (about 8%) has been achieved in SMA materials^42^, which gives us more possibility for unprecedented manipulations on electronic structure and property (our calculated results are shown in Figure S5).

## Methods

**Figure 1:**
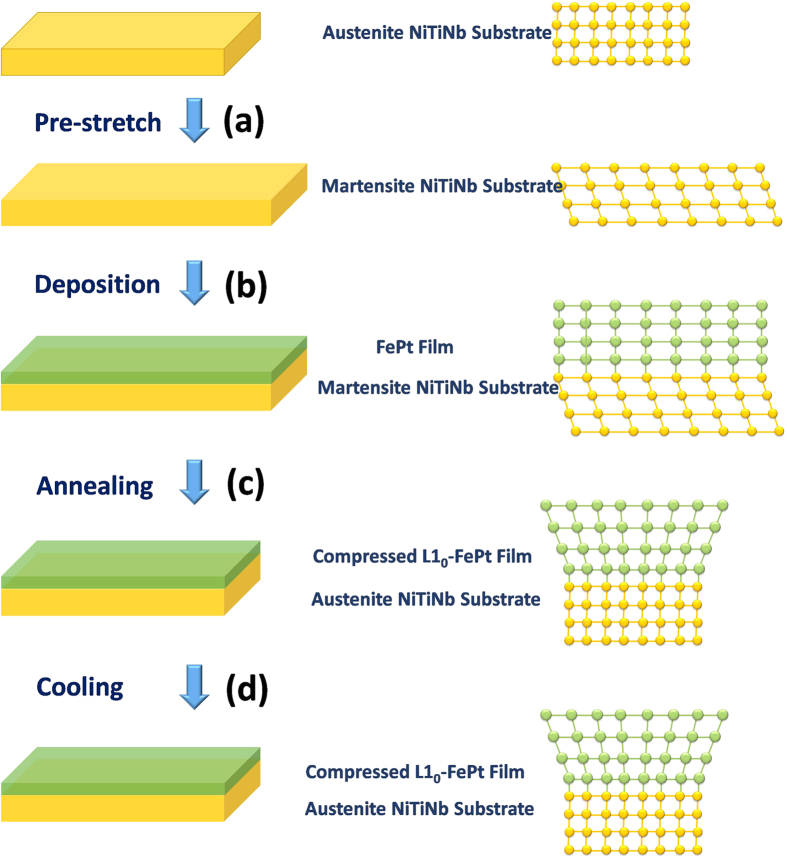
Schematics of sample preparation and corresponding atomic structure change during sample deformation. (**a**) Pre-treatment of NiTi(Nb) substrate: A NiTi(Nb) sheet was pre-stretched to induces reorientation of the martensitic phase. Then, the surface of the NiTi(Nb) sheet was cleaned and polished to achieve a surface roughness around 1 nm. (**b**) Film deposition: the FePt film was deposited on the polished SMA substrate by magnetron sputtering. (**c**) Sample annealing: the as-deposited sample was annealed to introduce both the L1_0_ ordering in the FePt film and the shape recovery in the substrate through an inverse martensitic phase transformation, resulting in a nonvolatile lattice strain in the L1_0_-FePt film. (**d**) Sample cooling: the compressive strain maintained in the film even after cooling down to room temperature.

**Figure 2:**
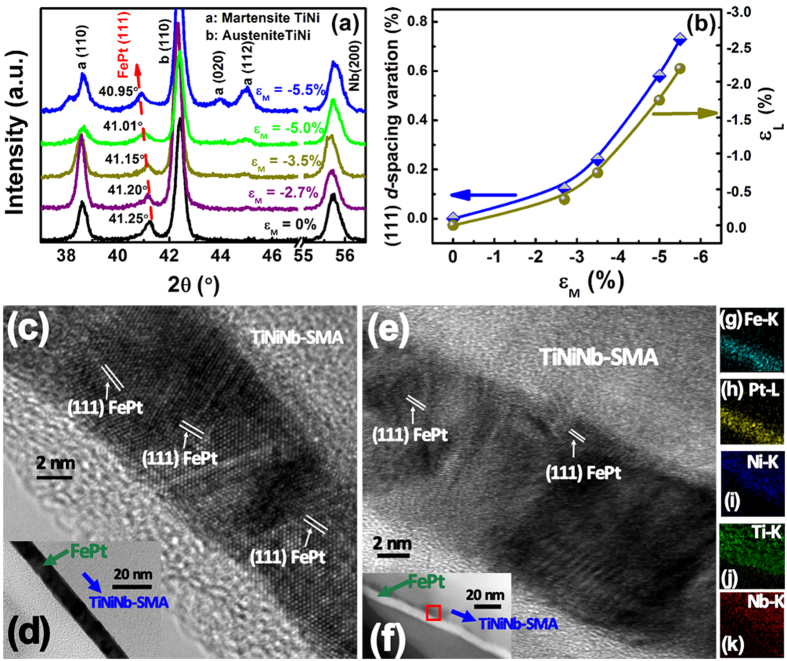
Microstructure and strain evolution in L1_0_-FePt(10 nm) films grown on shape memory alloy substrates with different amount of pre-deformation. (**a**) XRD patterns of the samples with different macro-strain (*ε*_M_) in the SMA substrates. (**b**)Variations of the FePt(111) *d*-spacing and the in-plane compressive lattice strain (*ε*_L_) with *ε*_M_. Cross-sectional HRTEM images of the samples: (**c,d**) *ε*_M_ = 0%; (**e,f**) *ε*_M_ = −3.5%. (**g–k**) Elemental mapping obtained from EDX scan of the area marked by the red square in Fig. 2f.

**Figure 3:**
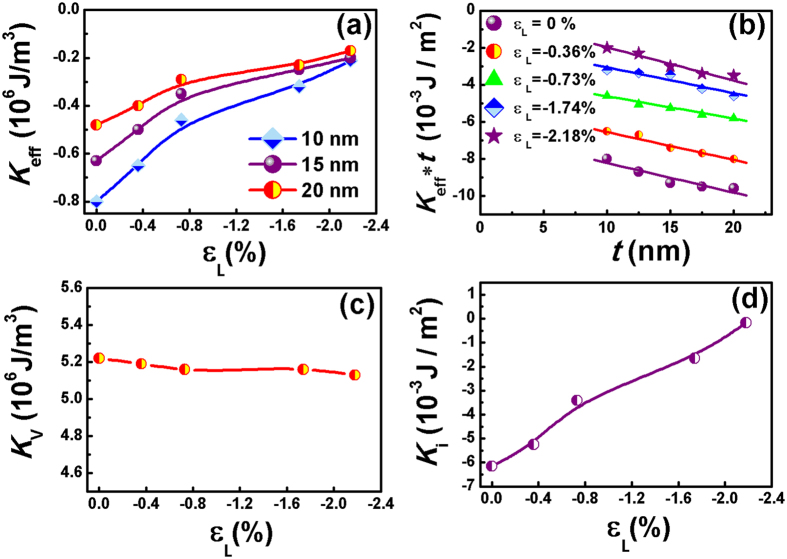
Magnetic property tunability induced by lattice strain in the L1_0_-FePt (*t *= 10, 15, 20 nm) films. (**a**) *K*_eff_ dependence on ε_L_ (from 0% to −2.18%). (**b**) Dependence of *K*_eff_*t on *t* as a function of strain. (**c,d**) Modification of bulk anisotropy (*K*_v_) and interfacial anisotropy (*K*_i_) by ε_L_, where *K*_v_ and *K*_i_ are obtained from the slope and the y axis interception of the curves in Fig. 3b, respectively.

**Figure 4:**
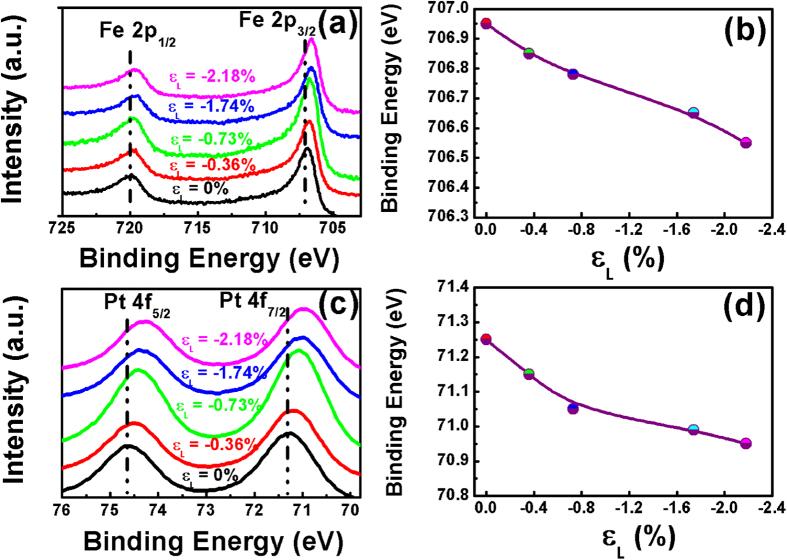
Effect of elastic strain on the interfacial electronic structure of the L1_0_-FePt film. (**a,c**) High resolution XPS spectra of characteristic Fe2p and Pt4f electrons at the NiTi(Nb)/FePt interface of the NiTi(Nb)-SMA/L1_0_-FePt (3 nm)/Ta(5 nm) sample; and (**b,d**) Binding energy evolutions of Fe2p_3/2_ and Pt4f_7/2_ electrons with different ε_L_.

Sample preparation process is shown in the schematic Fig. 1. (A) Preparation of NiTi(Nb) substrates. A cold rolled NiTi(Nb) sheet (0.5 mm thickness) was annealed at 400 °C for 20 min followed by an air cooling process. Then, the NiTi(Nb) sheet was cut into 3 mm × 100 mm pieces by electrical discharge machining. These pieces were then stretched in a tensile machine to introduce reorientation of martensitic phase in the sheets. The pre-loading amounts were chosen to be 6%, 8%, 10%, 12% which can produce revertible macro-strain (*ε*_M_) of 2.7%, 3.5%, 5.0%, 5.5%, respectively, as verified by dynamic thermomechanical analysis (DMA) curves (Figure S1). After unloading, the sheets were cut into our desirable size for film deposition and characterization. Finally, the surface of the sheets was cleaned and polished until the surface roughness is around 1 nm by using both rough grinding (choosing abrasive paper from 600 Mo to 3000 Mo in sequence) and elaborated polishing (using 5 μm, 1 μm and 0.05 μm alumina suspension as the polishing agent). (B) Film Deposition. FePt alloy films (3–20 nm) were deposited on the polished SMA substrates by magnetron sputtering with a base pressure of 5 × 10^−5^ Pa and an Ar working pressure of 0.45 Pa. The FePt film composition was confirmed to be Fe_52_Pt_48_ by inductively coupled plasma atomic emission spectrometry. (C) Annealing of samples. The as-deposited samples were annealed at 350 °C in a vacuum of 3 × 10^−5^ Pa for 20 min. Thermal treatment introduces both the L1_0_ ordering in the FePt film and shape recovery in the substrate through an inverse martensitic phase transformation. This process also introduces macro-strain transfer from the substrate to L1_0_-FePt film, constructing the nonvolatile lattice strain in the film. (D) Cooling the samples down to room temperature for subsequent characterizations.

**Figure 5:**
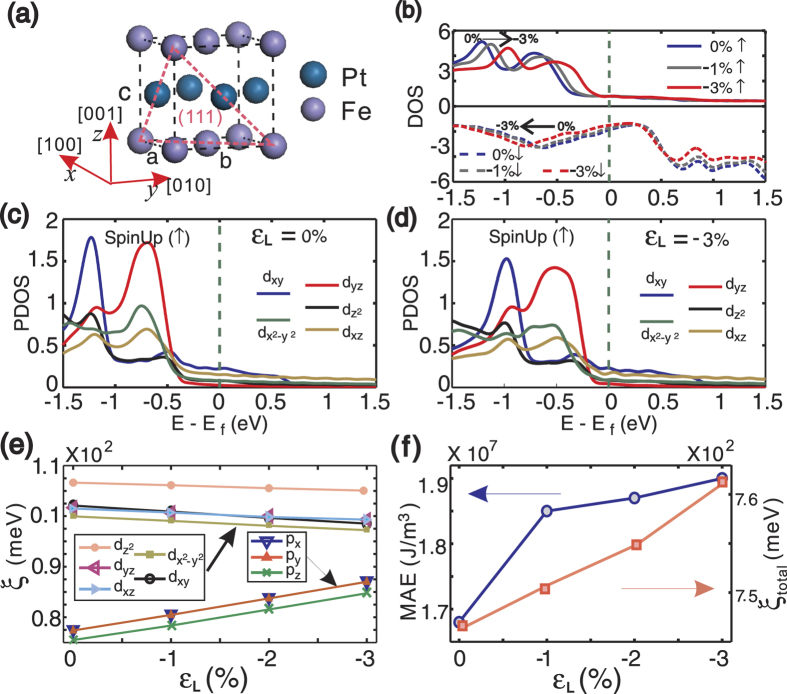
First-principles calculations on modifications of interfacial electronic structure and related physical properties due to the lattice strain in L1_0_-FePt. (**a**) Unstrained unit cell of L1_0_-FePt with lattice constants of a = b = 3.86 Å and c/a = 0.98. (**b**) Representative DOS of the spin-up (↑) and spin-down (↓) electrons in L1_0_-FePt films with three different strains. (**c,d**) Comparison of the PDOS of L1_0_-FePt films with and without a compressive lattice strain (ε_L_ = −3%). (**e**) Calculated SOC strengths (ξ) for different orbitals in L1_0_-FePt with a ε_L_ of −3%. (**f**) Dependences of total SOC strength (ξ _total_) and MAE of L1_0_-FePt on the ε_L_.

All of the property measurements and microstructure characterizations were conducted at room temperature. Magnetic properties were measured using a physical property measurement system (Quantum Design) with applied in-plane (along the strain) or out-of-plane fields up to 20kOe. The lattice strain development in the films was measured by XRD using Cu *Kα* radiation. The microstructure evolution after strain treatments was studied by the HRTEM (Tecnai F20). The electronic structure change at the NiTi(Nb)/FePt interface was revealed by the XPS measurements. Mg *K*_*α*_ radiation was used with the X-ray source that was run at 14.5 kV. The energy analyzer operated at a constant pass energy of 50 eV. Since the XPS detectable depth for Fe 2p and Pt 4f electrons are only 5.19 nm and 4.17 nm^43^, the NiTi(Nb)-SMA/L1_0_-FePt(3 nm)/Ta(5 nm) samples with different elastic strain treatments were designed for the XPS tests. The Ta protection layer was etched off with Ar^+^ ion before collecting XPS signals from the NiTi(Nb)/FePt interface with a detecting angle of 90°. The etching rate was about 0.25 Å/s. Our DFT calculations were carried out with the projector augmented plane wave (PAW) method^44^ and Perdew-Burke-Ernzerhof (PBE) exchange-correlation functional^45^, as implemented in the Vienna *ab* initio simulation package (VASP)^46^. For simplicity, it includes 2 Fe atoms and 2 Pt atoms per unit FePt cell. As shown in Fig. 5a, we took the experimental data for uncompressed system (*ε*_L_ =  0%), lattice constant a = b = 3.86 Å and c/a = 0.98. A fine K-mesh at 16 × 16 × 16 and energy cutoff at 500 eV were used to ensure numerical accuracy.

## Additional Information

**How to cite this article**: Feng, C. *et al.* Nonvolatile modulation of electronic structure and correlative magnetism of L1_0_-FePt films using significant strain induced by shape memory substrates. *Sci. Rep.* **6**, 20199; doi: 10.1038/srep20199 (2016).

## Supplementary Material

Supplementary Information
