# Corrigendum: Nonvolatile modulation of electronic structure and correlative magnetism of L1_0_-FePt films using significant strain induced by shape memory substrates

**DOI:** 10.1038/srep23878

**Published:** 2016-04-20

**Authors:** Chun Feng, Jiancheng Zhao, Feng Yang, Kui Gong, Shijie Hao, Yi Cao, Chen Hu, Jingyan Zhang, Zhongqiang Wang, Lei Chen, Sirui Li, Li Sun, Lishan Cui, Guanghua Yu

Scientific Reports 6: Article number: 2019910.1038/srep20199; published online: 02012016; updated: 04202016.

This Article contains typographical errors in in the Methods section.

“Mg *K*_*α*_ radiation was used with the X-ray source that was run at 14.5 kV. The energy analyzer operated at a constant pass energy of 50 eV. Since the XPS detectable depth for Fe 2p and Pt 4f electrons are only 5.19 nm and 4.17 nm^43^, the NiTi(Nb)-SMA/L1_0_-FePt(3 nm)/Ta(5 nm) samples with different elastic strain treatments were designed for the XPS tests.”

should read:

“Al *K*_*α*_ radiation was used with the X-ray source that was run at 14.5 kV. The energy analyzer operated at a constant pass energy of 50 eV. Since the XPS detectable depth for Fe 2p and Pt 4f electrons are only 3.9 nm and 3.2 nm^43^, the NiTi(Nb)-SMA/L1_0_-FePt(3 nm)/Ta(5 nm) samples with different elastic strain treatments were designed for the XPS tests.”

