# Supplementary material for: Nonvolatile modulation of electronic structure and correlative magnetism of L10-FePt films using significant strain induced by shape memory substrates
Source: Sci Rep. 2016 Feb 1;6:20199. doi: 10.1038/srep20199 (PMC4735331; doi:10.1038/srep20199)
Supplement: Supplementary Information [file srep20199-s1.pdf]

Supplementary Files

Nonvolatile modulation of electronic structure and correlative magnetism of $L1_0$-FePt films using significant strain induced by shape memory substrates

Chun Feng[1]*, Jiancheng Zhao[1]*, Feng Yang[2]*, Kui Gong[1,3], Shijie Hao[2], Yi Cao[1], Chen Hu[1], Jingyan Zhang[1], Zhongqiang Wang[2], Lei Chen[2], Sirui Li[2], Li Sun[4], Lishan Cui[2] & Guanghua Yu[1]

[1] *Department of Materials Physics and Chemistry, University of Science and Technology Beijing, Beijing 100083, People's Republic of China*. [2] *State Key Laboratory of Heavy Oil Processing, China University of Petroleum-Beijing, Beijing 102249, China*. [3] *Centre for the Physics of Materials and Department of Physics, McGill University, Montreal, Quebec H3A2T8, Canada*. [4] *Department of Mechanical Engineering and Texas Center for Superconductivity (TcSUH), University of Houston, Houston, Texas 77204, USA*.

Correspondence and requests for materials should be addressed to L.S. (lsun4@uh.edu) or L.S.C. (lscui@cup.edu.cn) or G.H.Y. (ghyu@mater.ustb.edu.cn)

* These authors contributed equally to this work.

Figure S1

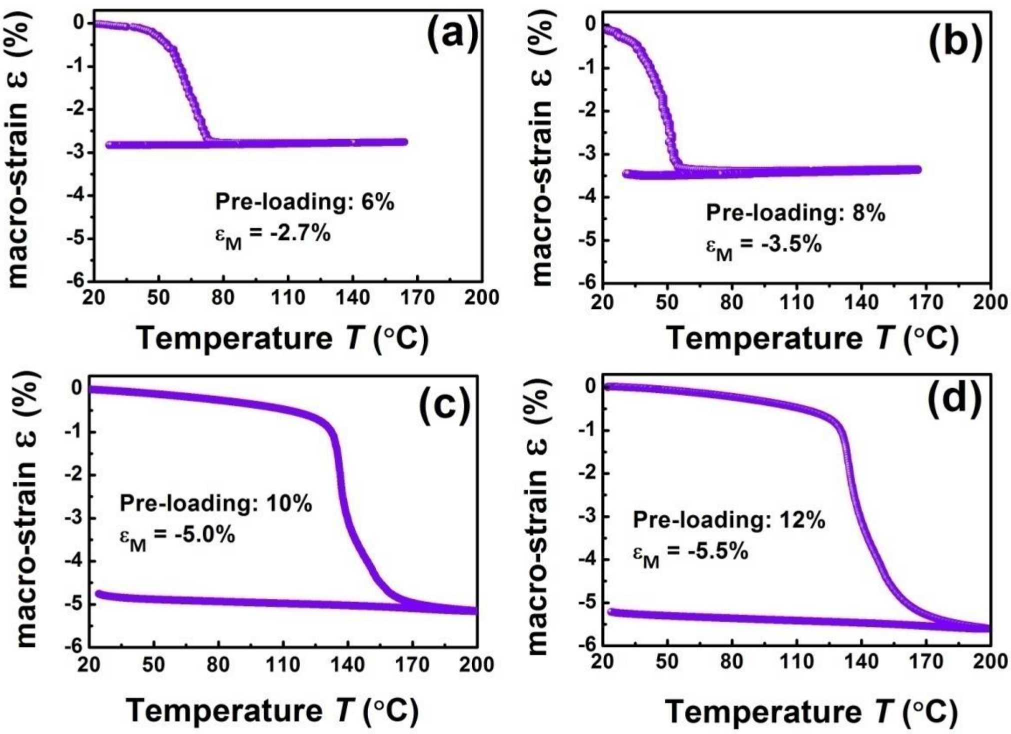

Figure S1 Dynamic thermomechanical analysis (DMA) curves of the pure SMA substrates. (a) Pre-loading 6%, recoverable macro-strain ε_M= -2.7%; (b) Pre-loading 8%, ε_M= -3.5%; (c) Pre-loading 10%, ε_M= -5.0%; (d) Pre-loading 12%, ε_M= -5.5%.

Figure S2

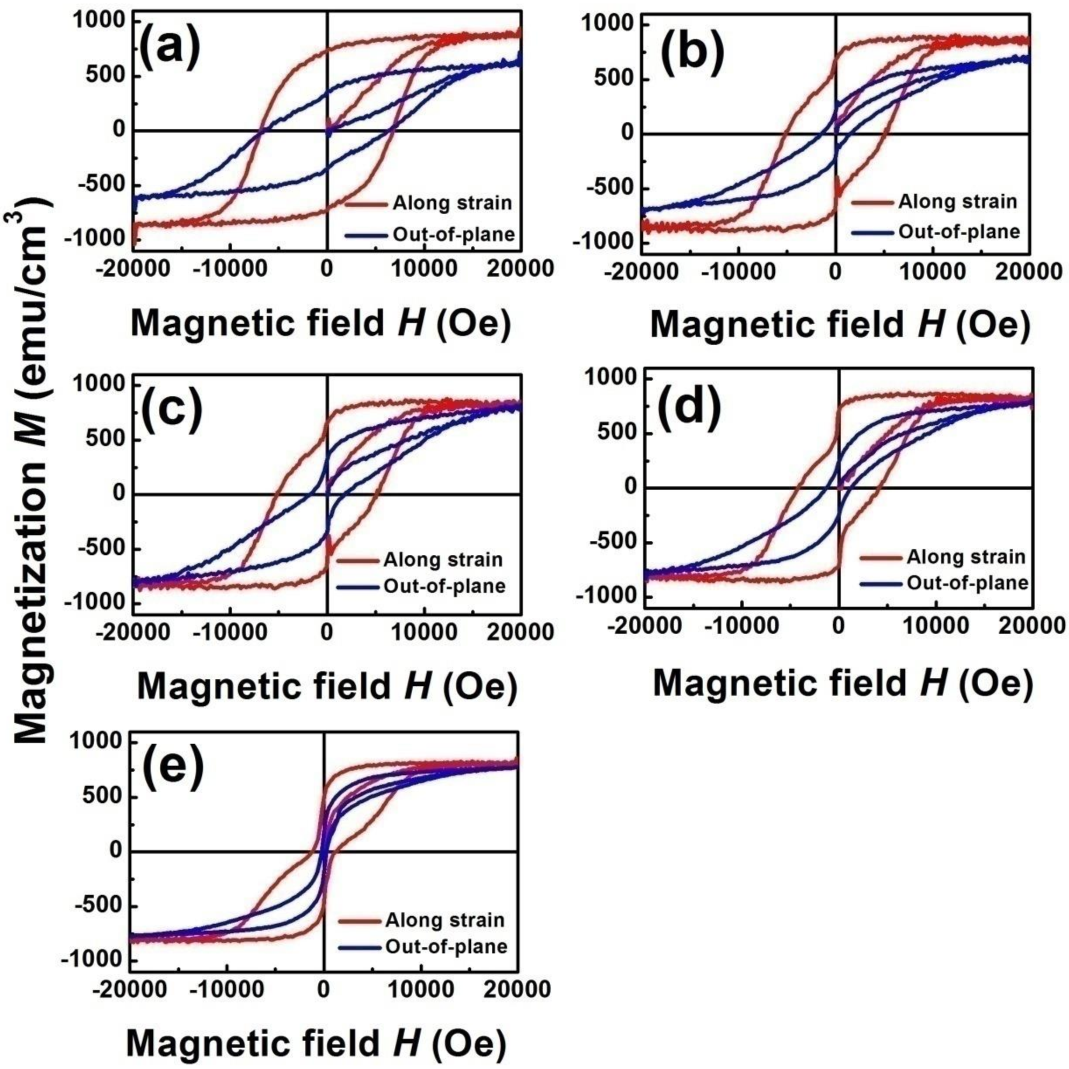

Figure S2 In-plane (along the strain) and out-of-plane hysteresis loops of the lattice strain treated $L1_0$-FePt(10 nm) film. (a) ε_L = 0%; (b) ε_L = -0.48%; (c) ε_L = -0.78%; (d) ε_L = -1.63%; (e) ε_L = -2.18%.

Figure S3

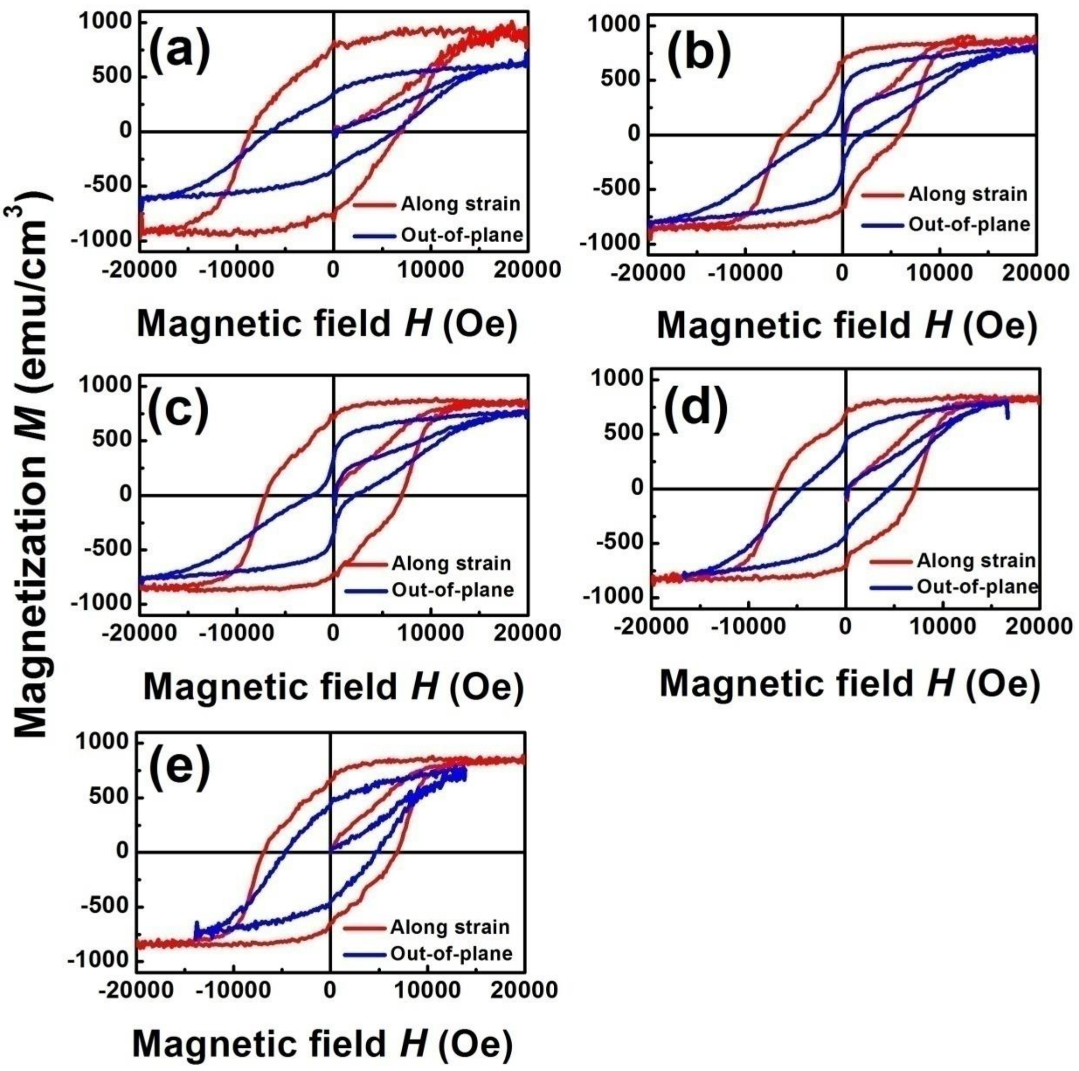

Figure S3 In-plane (along the strain) and out-of-plane hysteresis loops of the lattice strain treated $L1_0$-FePt(15 nm) film. (a) ε_L = 0%; (b) ε_L = -0.48%; (c) ε_L = -0.78%; (d) ε_L = -1.63%; (e) ε_L = -2.18%.

Figure S4

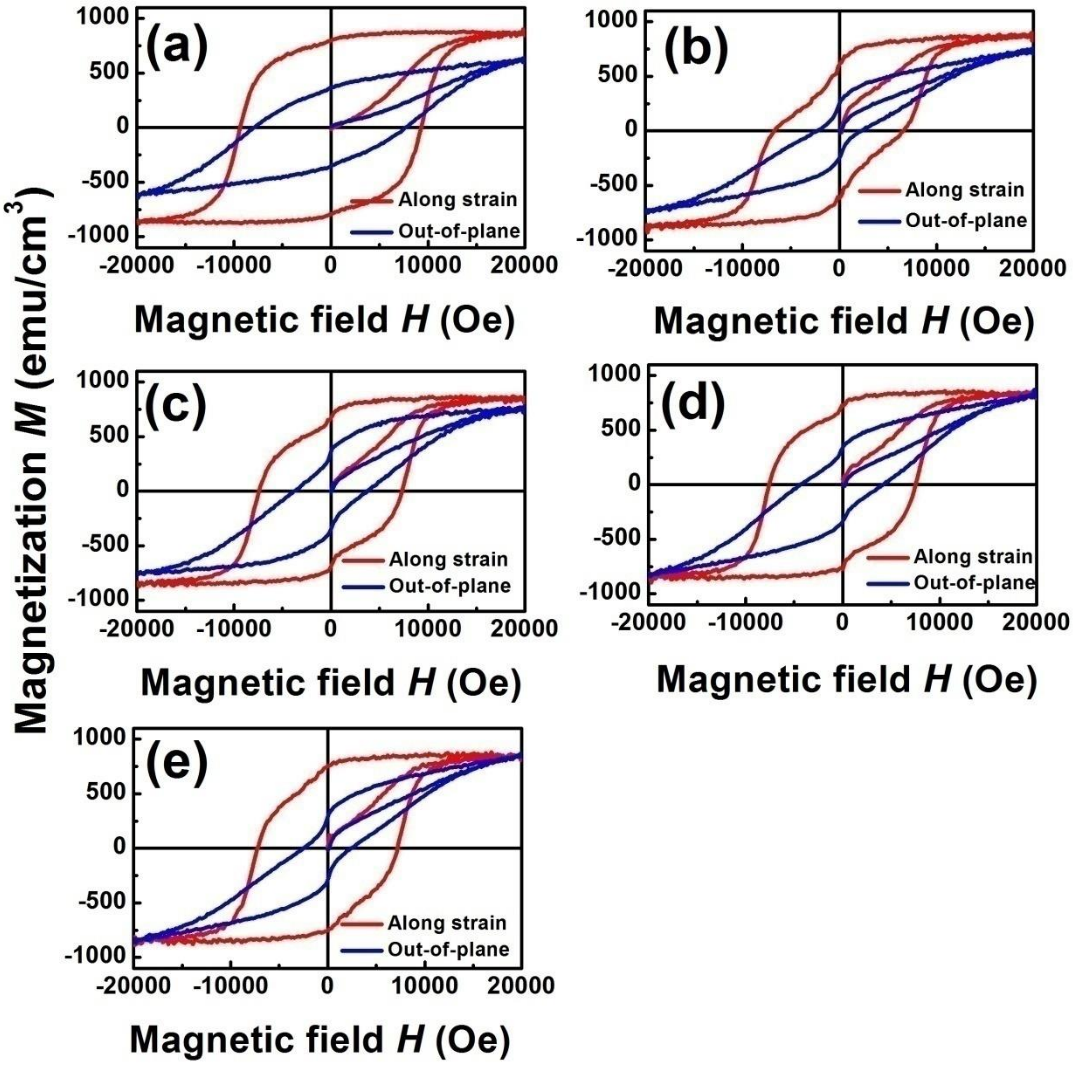

Figure S4 In-plane (along the strain) and out-of-plane hysteresis loops of the lattice strain treated $L1_0$-FePt(20 nm) film. (a) ε_L = 0%; (b) ε_L = -0.48%; (c) ε_L = -0.78%; (d) ε_L = -1.63%; (e) ε_L = -2.18%.

Figure S5

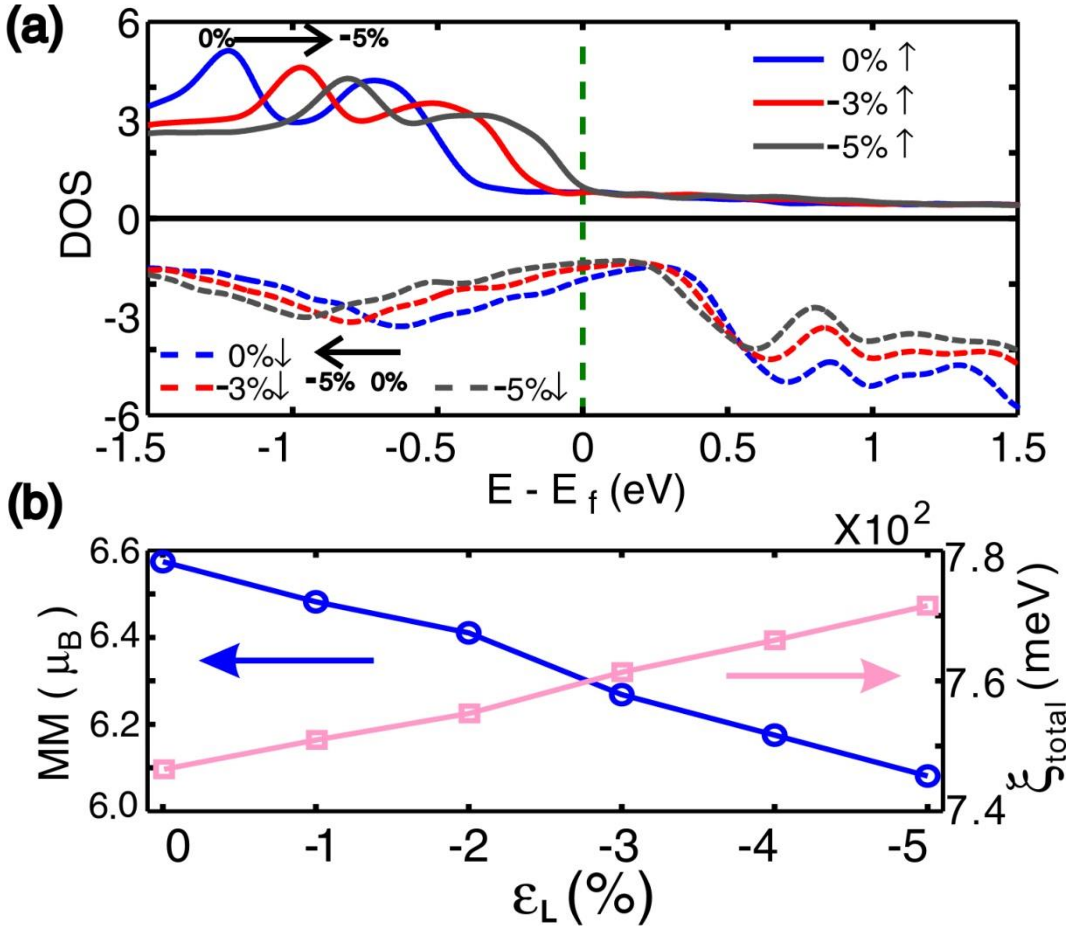

Figure S5 First-principles calculation results for larger strains ranging from 0% to 5%. (a) DOS of spin-up (↑) and spin-down (↓) electrons in $L1_0$-FePt films, the dashed line stands for Fermi level. When ε_L=-5%, the spin distribution shift by 0.5eV comparing with the ε_L=0% case. (b) Variations of magnetic moment (MM) and total SOC strength (ξ_{total}) with ε_L. The ξ_{total} increases from 746 meV (ε_L= 0%) to 769 meV (ε_L = -5%) with a considerable variation of 23 meV.

Figure S6

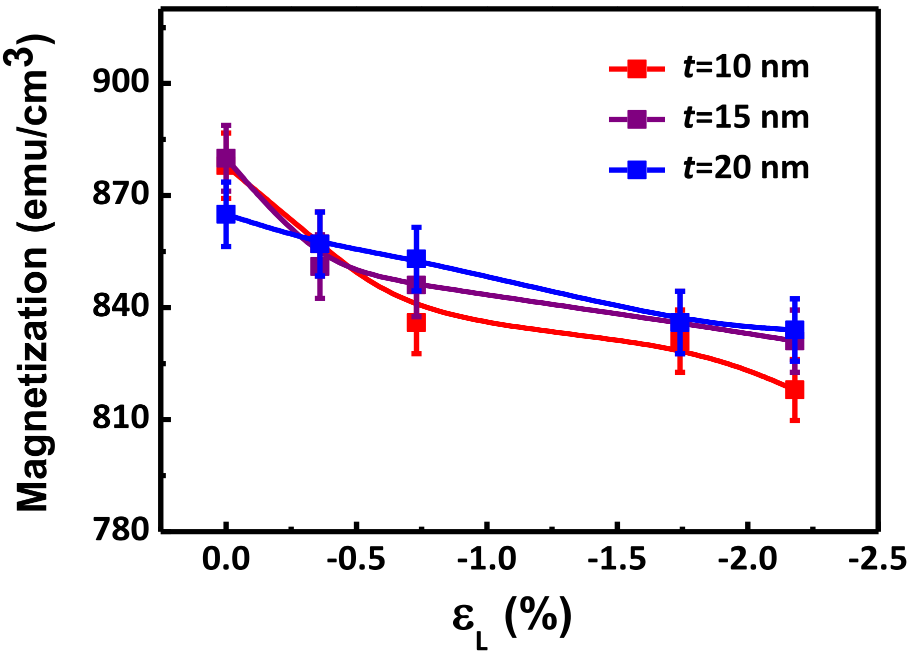

Figure S6 Variations of experimental magnetization in strain treated $L1_0$-FePt films with ε_L. *t* represents the thickness of the $L1_0$-FePt layer.
